# Supplementary material for: Cranial Neural Crest Specific Deletion of Alpl (TNAP) via P0-Cre Causes Abnormal Chondrocyte Maturation and Deficient Cranial Base Growth
Source: Int J Mol Sci. 2023 Oct 20;24(20):15401. doi: 10.3390/ijms242015401 (PMC10607232; doi:10.3390/ijms242015401)

Supplemental Figure S1. No differences in ISS between neonatal *Alpl*$^{fl/fl}$;*P0-Cre*$^{+}$ and control mice. Quantification of chondrocyte zones by H&E staining revealed no qualitative or quantitative differences between *Alpl*$^{fl/fl}$;*P0-Cre*$^{+}$ as compared to *Alpl*$^{fl/fl}$ mice. Red = *Alpl*$^{fl/fl}$, Blue = *Alpl*$^{fl/fl}$; *P0-Cre*$^{+}$. Zones were demarcated according to the following criteria. RZ: The nucleus is round. PZ: The nucleus is flat. PHZ: Adjacent to the PZ, and the cell shape is flattened and enlarged. HZ: The shape is greatly enlarged.

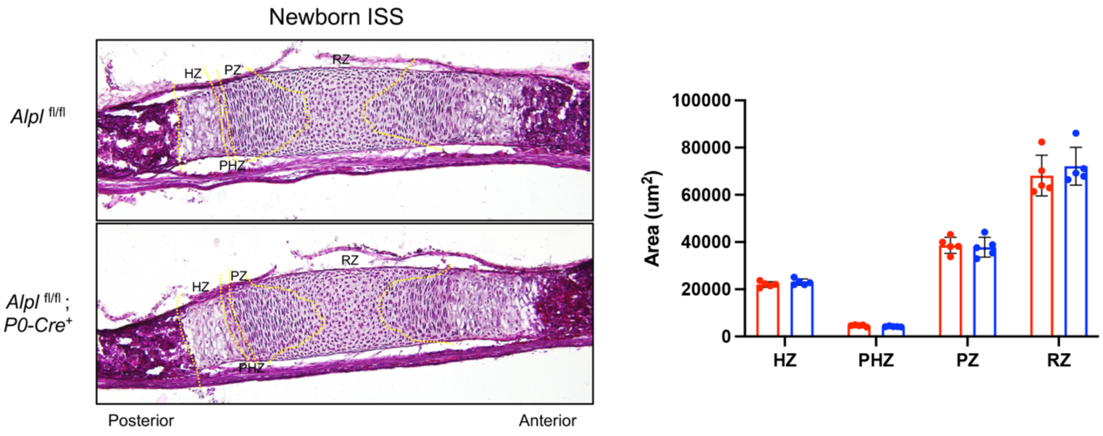

Supplemental Figure S2. Col II expression is not altered in the ISS of *Alpl*$^{fl/fl}$;*P0-Cre*$^{+}$ mice. Quantification of immunofluorescent staining for collagen 2 revealed no differences in any chondrocyte zones of *Alpl*$^{fl/fl}$; *P0-Cre*$^{+}$ as compared to *Alpl*$^{fl/fl}$ mice. Yellow scale bars = 200 μm. Red = *Alpl*$^{fl/fl}$, Blue = *Alpl*$^{fl/fl}$; *P0-Cre*$^{+}$. Zones were demarcated according to the following criteria. RZ: The nucleus is round. PZ: The nucleus is flat. PHZ: Adjacent to the PZ, and the cell shape is flattened and enlarged. HZ: The shape is greatly enlarged.

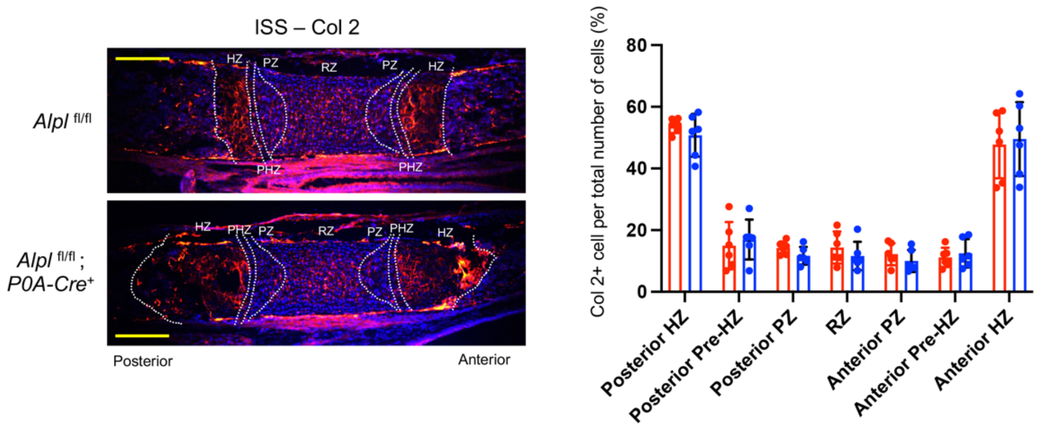

Supplemental Figure S3. Landmarks used for whole skull and cranial base linear distance measurements. (A) Craniofacial skeletal landmarks: 1 = nasale, 2 = nasion, 3 = bregma, 4 = pari, 5,6 = joining of squamosal body to zygomatic process of squamous portion of temporal bone, 7 = paro, 8,9 = intersection of frontal process of maxilla with frontal and lacrimal bones (inner canthal distance), 10 = inferior portion of the spheno-occipital synchondrosis. **(B)** Illustration of the cranial base: PS presphenoid bone; BS = basisphenoid bone; BO = basioccipital bone; SOS = spheno-occipital synchondrosis; ISS = intersphenoid synchondrosis.

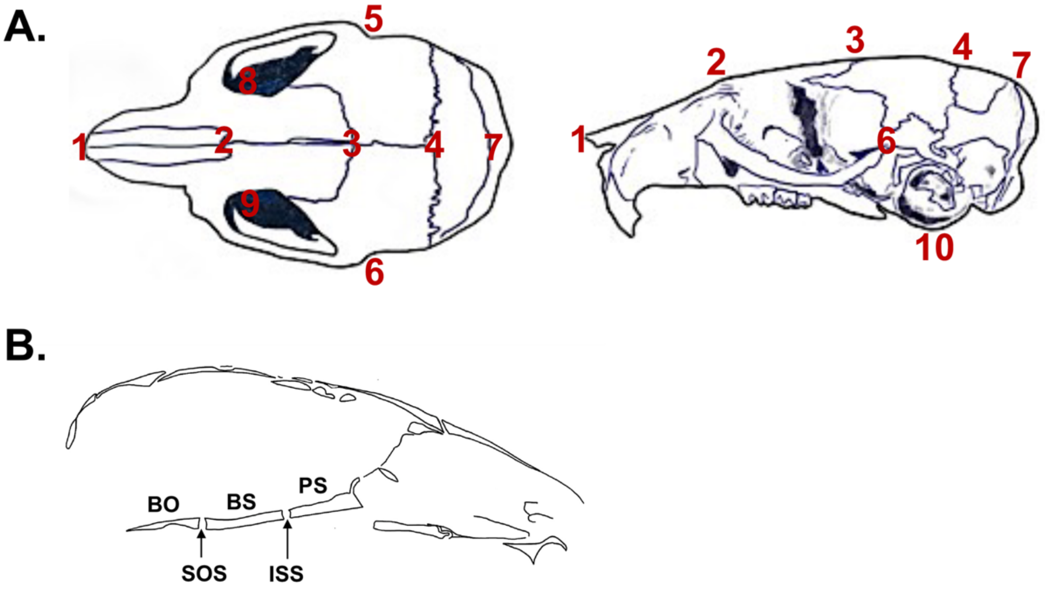

Supplemental Figure S4. ***Alpl*** **deletion by P0-Cre in cranial neural crest cells does not alter hypertrophic zone widths in the spheno-occipital (SOS) synchondrosis at postnatal day 5. (A).** Hematoxylin and Eosin staining showing the histology of the SOS with/without ablation of *Alpl* in cranial neural crest derived cells. No differences in chondrocyte zone widths raw, or when normalized to total SOS length are seen. **(B)** Alcian blue staining for cartilaginous glycosaminoglycans in the SOS of *Alpl*$^{fl/fl}$ and *Alpl*$^{fl/fl}$; *P0-Cre*$^{+}$ mice is shown. No differences in alcian blue staining are seen. n=6 per genotype. Black scale bars = 200 µm. Red = *Alpl*$^{fl/fl}$, Blue = *Alpl*$^{fl/fl}$; *P0-Cre*$^{+}$. Zones were demarcated according to the following criteria. RZ: The nucleus is round. PZ: The nucleus is flat. PHZ: Adjacent to the PZ, and the cell shape is flattened and enlarged. HZ: The shape is greatly enlarged.

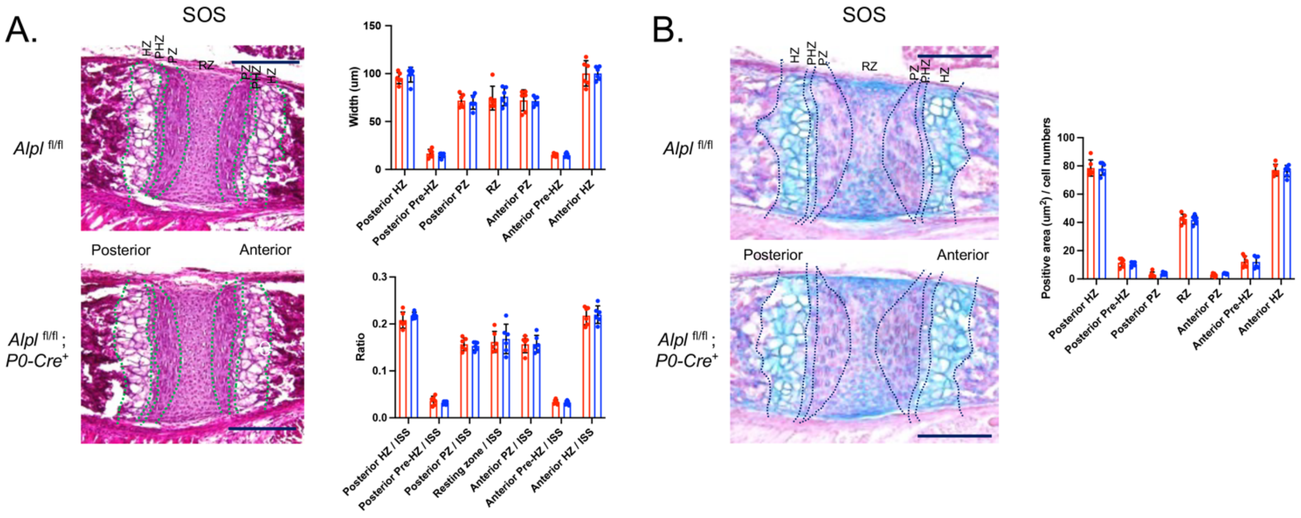

Supplemental Figure S5. *Alpl* deletion by P0-Cre in cranial neural crest cells does not alter expression of PTHrP, IHH, Sox9 or ColX in the SOS at postnatal day 5. All stains include DAPI nuclear blue counterstain. **(A)** Cells were immunostained for PTHrp (red), then quantified by cell count as normalized to total cell number per chondrocyte zone. Quantification shows no differences in PTHrp expression between *Alpl*$^{fl/fl}$;*P0-Cre*$^{+}$mice and control mice. **(B)** Cells were immunostained for IHH (red), then quantified by cell count as normalized to total cell number per chondrocyte zone. Quantification shows no differences in IHH expression between *Alpl*$^{fl/fl}$;*P0-Cre*$^{+}$mice ando control mice. **(C)** Cells were immunostained for Sox9 (red), then quantified by cell count as normalized to total cell number per chondrocyte zone. Quantification shows no differences in Sox9 expression between *Alpl*$^{fl/fl}$;*P0-Cre*$^{+}$ and control mice. **(D)** Cells were immunostained for ColX (red), then quantified by cell count as normalized to total cell number per chondrocyte zone. Quantification shows no differences in ColX expression between *Alpl*$^{fl/fl}$;*P0-Cre*$^{+}$ and control mice. n=6 per genotype. Yellow scale bars = 200 µm. Red = *Alpl*$^{fl/fl}$, Blue = *Alpl*$^{fl/fl}$;*P0-Cre*$^{+}$mice.

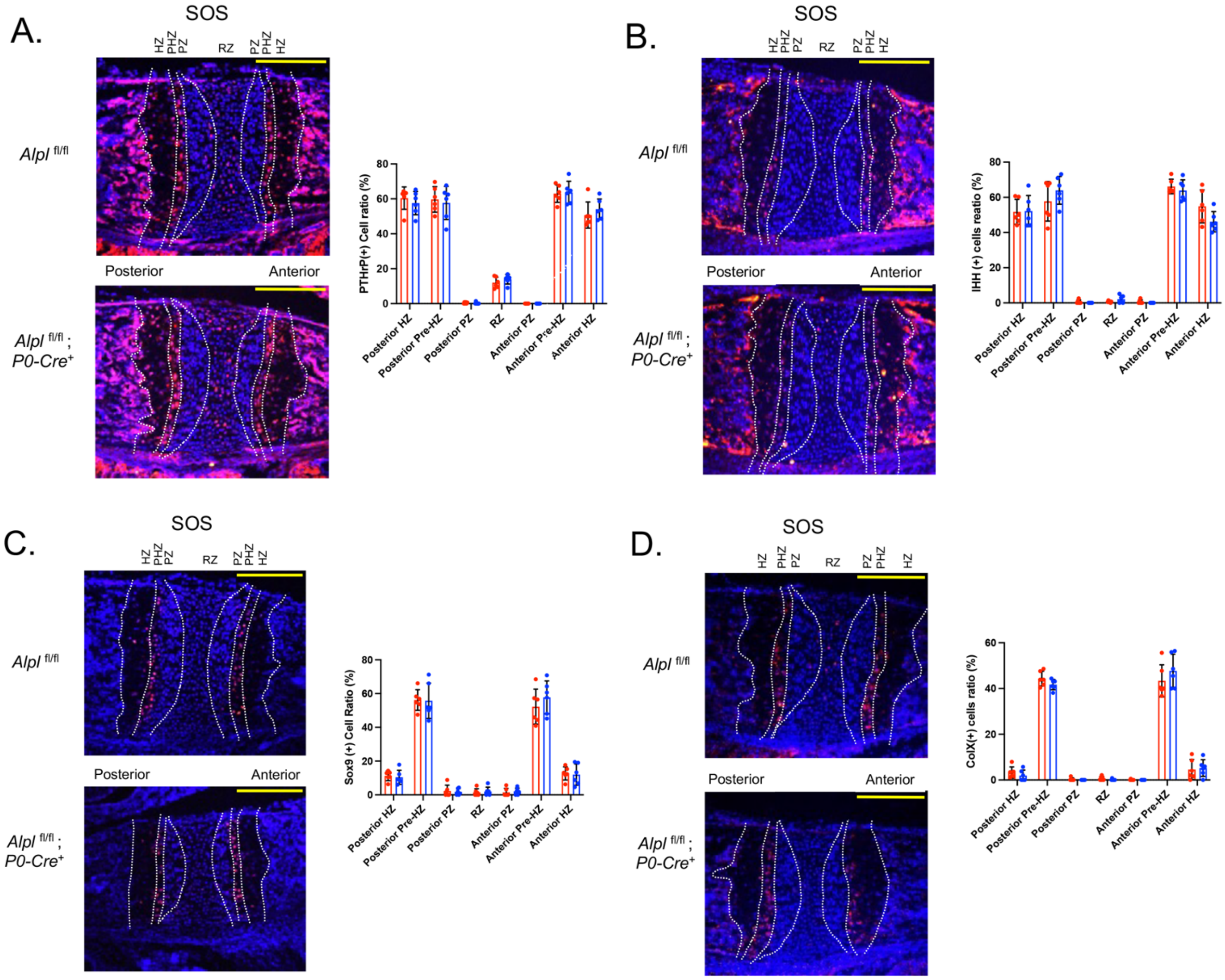

Supplement: Supplementary file 1 [file ijms-24-15401-s001.zip › ijms-2604949-supplementary.pdf]
